# A 4-Benzene-Indol Derivative Alleviates LPS-Induced Acute Lung Injury Through Inhibiting the NLRP3 Inflammasome

**DOI:** 10.3389/fimmu.2022.812164

**Published:** 2022-02-10

**Authors:** Junmei Li, Yang Bai, Yiting Tang, Xiangyu Wang, María José Cavagnaro, Ling Li, Zhaozheng Li, Yi Zhang, Jian Shi

**Affiliations:** ^1^Department of Hematology and Critical Care Medicine, The Third Xiangya Hospital, Central South University, Changsha, China; ^2^Department of Physiology, School of Basic Medical Science, Central South University, Changsha, China; ^3^College of Medicine-Phoenix, University of Arizona, Phoenix, AZ, United States; ^4^Department of Gastrointestinal Surgery, The Third Xiangya Hospital, Central South University, Changsha, China; ^5^Department of Spine Surgery, The Third Xiangya Hospital, Central South University, Changsha, China

**Keywords:** sepsis, acute lung injury, NLRP3 inflammasome, 4-benzene-indol derivative, pharmaceutical target, inflammatory cytokines, critical care medicine, basic and clinic immunology

## Abstract

Acute lung injury (ALI) is a common complication of critical illness that could frequently lead to acute respiratory distress syndrome and other serious clinical consequences. Sepsis is one of the major and most common inducements among all causes of ALI. Due to its high incidence and mortality rate and also the complexity in treatment, sepsis-related ALI has become an urgent clinical problem waiting to be solved effectively. At present, only the protective ventilation strategy, restrictive fluid management, and antibiotics application are measures that can improve the prognosis with evidence-based medical proof. No pharmacological treatment is currently available to protect or significantly reverse the prognosis. Seeking for effective interventions measures for sepsis-related ALI is one of the most necessitous research directions. In this research, a conspicuous discovery of treatment-related translational use for a 4-benzene-indol derivative was elaborated by screening a large number of chemical compounds. The results showed that 4-benzene-indol derivative could not only suppress the activation of NLRP3 inflammasome both *in vitro* and alleviate LPS-induced ALI *in vivo* but also suppress the NLRP3 inflammasome in human myeloid leukemia mononuclear cells (THP-1) cell lines. Mechanistically, 1,2-diol blocks the NLRP3 inflammasome activation by disrupting NLRP3–NEK7 interaction and the subsequent NLRP3 inflammasome assembly and activation. To summarize, this research indicated that the newly-discovered 4-benzene-indol derivative targets NLRP3 inflammasome signaling, which consequently alleviates sepsis-related ALI. Collectively, the 4-benzene-indol derivative may serve as a potential therapeutic drug and NLRP3 inflammasome signaling would be a novel pharmaceutical target for clinical treatment of sepsis-related ALI.

## Introduction

As a life-threatening multiple organ dysfunction attributable to maladjusted host immune responses to infection, sepsis is usually the common pathway to serious prognosis such as systemic inflammatory response syndrome (SIRS), multiple organ dysfunctions (MODS) and a high risk of death (1–3). In sepsis-induced MODS, the lung is the most vulnerable organ (4), several studies have demonstrated that more than 50% of patients with sepsis will eventually become acute lung injury (ALI) or acute respiratory distress syndrome (ARDS) (5, 6), which characterized by acute onset noncardiogenic pulmonary edema and hypoxemia (7). Despite the considerable efforts to find potential treatment strategies, the protective ventilation strategy, restrictive fluid management, and antibiotics application are the only available treatment options for patients with sepsis and ALI. Nevertheless, these measures have limited effect on decreasing the high mortality rate from sepsis (8, 9).

Recently, multiple studies have highlighted the crucial component of the NLR family pyrin domain containing 3 (NLRP3) inflammasome in sepsis (10, 11). As a multiple-protein complex, NLRP3 inflammasome is composed by NLRP3, apoptosis-associated speck-like protein containing a CARD (ASC) and Caspase-1 which are known as innate immune sensors (12). The assembly of NLRP3 inflammasome will lead to the activation of Caspase-1, and Caspase-1 will then promote the cleavage of GSDMD and also pro-IL-1β and pro-IL-18. Hence, NLRP3 inflammasome plays an essential role in inflammation and innate immunity (13). Production of proinflammatory cytokines interleukin such as IL-1β and IL-18 and NLRP3 inflammasome in macrophages, jointly result in the pyroptosis process of macrophages during ALI (14–16). Moreover, several inhibitors of NLRP3 inflammasome have been demonstrated to exert beneficial effects for ALI in animal model tests which suggest that NLPR3 inflammasome might be a novel potential pharmaceutical target for the clinical treatment of ALI (17, 18).

In this study, by screening a large number of chemical compounds, we identified 4-[2-(1H-indol-3-yl)-1,3-thiazol-4-yl] benzene-1,2-diol (1,2-diol), a 4-benzene-indol derivative, suppressed NLRP3 inflammasome activation both *in vitro* and alleviates LPS-induced ALI *in vivo*. Mechanistically, 1,2-diol blocks the NLRP3 inflammasome activation by disrupting NLRP3-NEK7 interaction and the subsequent NLRP3 inflammasome assembly and activation. Furthermore, 1,2-diol can also suppress the NLRP3 inflammasome in human myeloid leukemia mononuclear cells (THP-1) cell lines. Collectively, this study provides a new avenue for LPS induced ALI, which may facilitate the therapeutic strategy of sepsis and ALI.

## Materials and Methods

### Animals

C57BL/6 mice (8–10 weeks old) weighing 25–28 g were purchased from the Hunan SJA Laboratory Animal Co. Ltd. (Changsha, China). *NLRP3^−/−^* mice were provided by Professor Rongbin Zhou. Mice were kept under specific pathogen-free (SPF) conditions. All animal experiments were approved by the Institutional Animal Care and Use Committee of Central South University.

### Cell Culture

THP-1 cells were obtained from American Type Culture Collection (Manassas, VA). Primary peritoneal macrophages from C57BL/6 mice were isolated by peritoneal lavage with 10 ml RPMI 1640 medium (Gibco™ |Thermo Fisher Scientific).THP-1 cells and primary peritoneal macrophages were cultured in RPMI 1640 medium which was supplemented with 10% FBS (Gibco™ |Thermo Fisher Scientific) and 1% penicillin/streptomycin at 37°C in a humidified incubator of 5% CO_2_.

### Inflammasome Activation

Primary peritoneal macrophages from C57BL/6 mice or THP1 cells were seeded in 24-well (5 × 10^5^) or 6-well (2 × 10^6^) culture plates. The next day, we removed medium and primed macrophages with LPS (InvivoGen tlrl-3pelps,100 ng/ml) for 3 h followed by 1,2-diol for 1 h. At the last, we treated macrophages with stimulation as follows: ATP (InvivoGen tlrl-atp, 5 mmol/L) or nigericin (InvivoGen tlrl-nig, 10 μmol/L) for 1 h and MSU (InvivoGen tlrl-msu, 200μg/ml) or SiO_2_ (InvivoGen tlrl-sio, 20 μg/ml) for 6 h. THP-1 cells should be treated with 100 ng/ml PMA for 12 h before seeding in culture plates.

### ELISA Assay for Cytokines

ELISA kits were used to detect the mouse IL-1β (eBioscience), TNF-α (eBioscience), IL-6 (eBioscience), and human IL-1β (Biolegend, #437004), human TNF-α (eBioscience) according to the manufacturer’s instructions.

### ASC Speck Formation

Primary peritoneal macrophages were seeded in chamber slides and the following day, the cells were given with indicated stimuli. Then we used 4% Paraformaldehyde (PFA) to fix the cells for 15 min at room temperature. Then the cells were washed with PBS, permeabilized with 0.1% Triton X-100, and blocked with PBS buffer which were containing 3% BSA. Then we used the anti-ASC (Adipogen AL177, 1:200 at 4°C overnight) to incubate with the cells and used DyLight 488-labeled as the secondary antibody (1:50 at room temperature for 45 min). Finally, DAPI was used to stain nuclei and cells were visualized by fluorescence microscope (Nikon Ti2-U).

### Immunoprecipitation and Western Blot

Peritoneal macrophages which were treated with stimulation were lysed in immunoprecipitation (IP) buffer, namely, a protease inhibitor cocktail, and then the cell lysates were incubated overnight at 4°C with the specific antibodies. The next day, immunoprecipitates were washed by IP buffer 4 times and incubated with Protein A/G plus-agarose(Santa Cruz sc-2003) at 4°C for 4 h. At last, immunoprecipitates were boiled with 1% (w/v) SDS sample buffer. The proteins bound by antibody were precipitated by protein A/G beads and subjected to immunoblotting analysis. The proteins were separated by SDS-PAGE, and then we have translated the proteins onto PVDF membranes for immunoblot analysis. After that, the membranes were blocked with 5% dried milk in TBS-T (50 mM Tris/HCL, 150 mM NaCl, pH 7.6 and 0.1% Tween-20) for 1 h at room temperature. After blocking, PVDF membranes were incubated with various primary antibodies such as human IL-1β (Abcam ab9722), human GSDMD (Abcam ab210070), Caspase-1 (Abcam ab179515), IL-1β (RD systems AF-401-NA), NLRP3 (Adipogen), ASC (Adipogen AL177), GSDMD (Abcam ab133514) and NEK7 (Abcam ab133514) at 4°C overnight. The next day, membranes were washed with TBS-T and incubated in corresponding horseradish peroxidase-conjugated secondary antibodies (1:5,000, KPL) for 1 h at room temperature.

### ASC Oligomerization

Peritoneal macrophages were treated with indicated stimuli, then the cells were lysed with Triton buffer [50 mM Tris–HCl (pH 7.5), 50 mM NaCl, 0.5% Triton X-100], 0.1 mM phenylmethylsulfonyl fluoride (PMSF) and EDTA-free protease inhibitor cocktail at 4°C for 10 min. The lysates were centrifuged at 6,000*g* at 4°C for 15 min, the supernatant was collected and pellets were washed twice and re-suspended in 200 μl Triton buffer, then added 2 mM disuccinimidyl suberate (DSS) and cross-linked at 37°C for 30 min. Samples were centrifuged and the pellets were dissolved in sodium dodecyl sulfate (SDS) loading buffer for western blotting.

### Intracellular K+ Level

Peritoneal macrophages were treated with indicated stimuli, then the medium was removed and cells were washed with PBS 2 times, and then cells were soaked with 0.9% NS for 15 min. We scraped cells and collected them to count under the microscope. At last, cells were added 0.9% NS so that its concentration becames 1× 10^6^, and then tested the K^+^ level by Hitachi 7600 automatic biochemical analyzer (Hitachi Coro, Tokyo, Japan).

### ALI and Endotoxemia Model

For ALI model, C57BL/6 mice were anesthetized using 1% Pentobarbital administered intraperitoneally and mice were injected intraperitoneally (i.p) with 40 mg/kg 1,2-diol or DMSO 30 min before intratracheally (i.t) instilled with 15 mg/kg LPS (*E. coli* O111:B4; Sigma). After 48 h, we killed the mice, harvested the lung tissue and collected the bronchoalveolar lavage fluid. For endotoxemia model, C57BL/6 mice were injected intraperitoneally with 40 mg/kg 1,2-diol or DMSO 30 min before i.p. with 25 mg/kg LPS. After 16 h, we killed the mice, harvested the lung tissue and collected the serum.

### Histology

The lung tissue sections from mouse were fixed in 4% PFA and embedded by paraffin. Tissue sections were deparaffinized and rehydrated. The sections were prepared and stained with H&E using standard procedures. Slides were examined under a Nikon ECL IPSE Ci biological microscope, and images were captured with a Nikon DS-U3 color digital camera.

### Statistical Analysis

All data were analyzed using GraphPad Prism software (version 8.2). Data were analyzed using by student’s t-test and were used for comparison between two groups or one-way ANOVA followed by *post-hoc* Bonferroni test for multiple comparisons. A p-value <0.05 was considered statistically significant for all experiments. All values are presented as the mean ± SD.

## Results

### 1,2-Diol Blocks NLRP3 Activation in Mouse Peritoneal Macrophages

**Figure 1:**
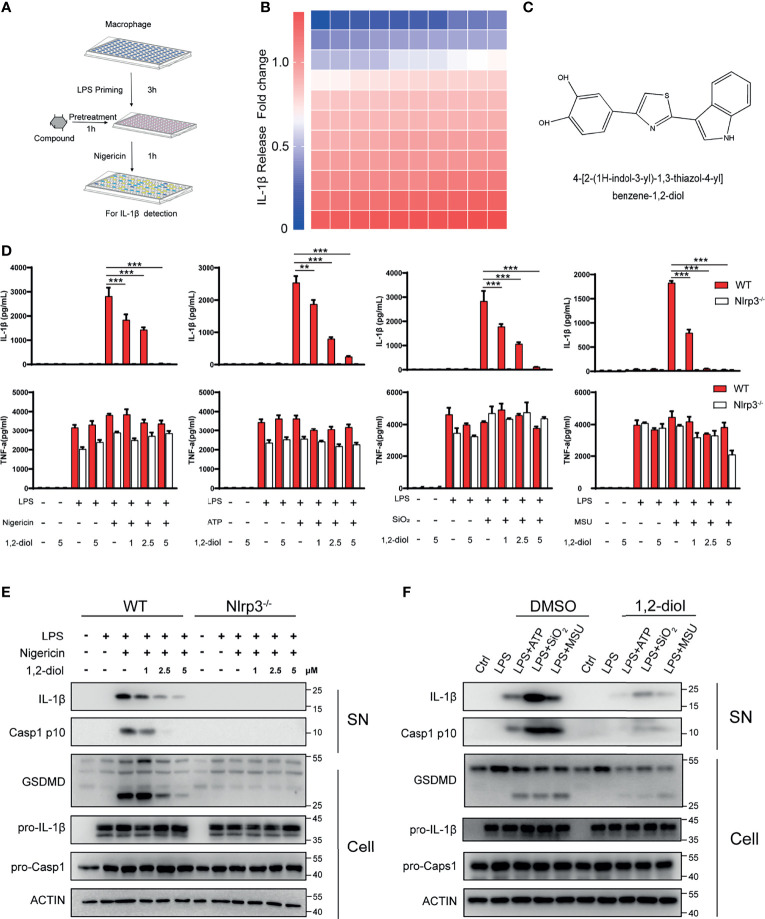
1,2-Diol blocks NLRP3 activation in mouse peritoneal macrophages.**(A)** Schematic diagram of the screening steps from the compound library in mouse macrophage stimulated with nigericin and LPS. **(B)** Heatmap of IL-1β release changes in LPS-primed primary macrophages treated with 5 μM inhibitors and then stimulated with nigericin with 110 bioactive compounds library. **(C)** Chemical structure of 4-[2-(1H-indol-3-yl)-1,3-thiazol-4-yl]benzene-1,2-diol. **(D)** cytokines (IL-1β and TNFα) release from WT or Nlrp3^−/−^ peritoneal macrophages primed by LPS and stimulated with nigericin, ATP, SiO_2_ or MSU in the absence or not of 1,2-diol with different doses. **(E)** Western blots for Caspase-1 and IL-1β in the supernatant or the cleavage of GSDMD, the expression of pro-Caspase-1 and pro-IL-1β in the cell lysates of WT or Nlrp3^−^/^−^ peritoneal macrophages primed by LPS and stimulated with nigericin in the absence or not of 1,2-diol with different doses. **(F)** Western blots for Caspase-1 and IL-1β in the supernatant or the cleavage of GSDMD, the expression of pro-Caspase-1 and pro-IL-1β in the cell lysates of peritoneal macrophages primed by LPS and stimulated with ATP, SiO2 or MSU in the absence or not of 1,2-diol with 5 μM dose. Graphs show the mean ± SD of technical replicates and are representative of at least three independent experiments. (**p < 0.01; ***p < 0.001).

To discover a potential approach for the treatment of NLRP3-driven diseases, we screened NLRP3 inflammasome inhibitors in a bioactive compound library purchased from Selleck. We treated mouse peritoneal macrophages with nigericin and LPS to activate NLRP3 inflammasome and detected IL-1β release (**Figure 1A**). After screening 110 compounds, we identified 4-[2-(1H-indol-3-yl)-1,3-thiazol-4-yl]benzene-1,2-diol (1,2-diol), a novel indole-based small molecule compound, as the most potent inhibitor for NLRP3 inflammasome (**Figures 1B, C**). The results showed that 1,2-diol exhibited dose-dependent inhibitory effects on NLRP3-dependent IL-1β secretion, but had no effects on inflammasome-independent cytokine TNF-α production (**Figure 1D**). As revealed by western-blot, 1,2-diol significantly attenuated Caspase-1 and GSDMD cleavage, the release of IL-1β, but did not inhibit the precursors of IL-1β and Caspase-1 expression (**Figure 1E**). To further verify the inhibitory effect of 1,2-diol on NLRP3 inflammasome, we used other NLRP3 agonists, such as monosodium urate crystals (MSU), ATP or silicon crystals (SiO_2_).The results showed 1,2-diol can also abolish the secretion of IL-1β, but had no effects on inflammasome-independent cytokine TNF-α production (**Figure 1D**). The cleaved of Caspase-1,GSDMD and the release of IL-1β were impaired, and also the precursors of IL-1β and Caspase-1 expression did not affect either (**Figure 1F**). Thus, these results suggest that 1,2-diol can be an inhibitor of NLRP3 inflammasome.

### 1,2-Diol Suppresses NLRP3 Inflammasome Activation in THP-1 Cells

**Figure 2:**
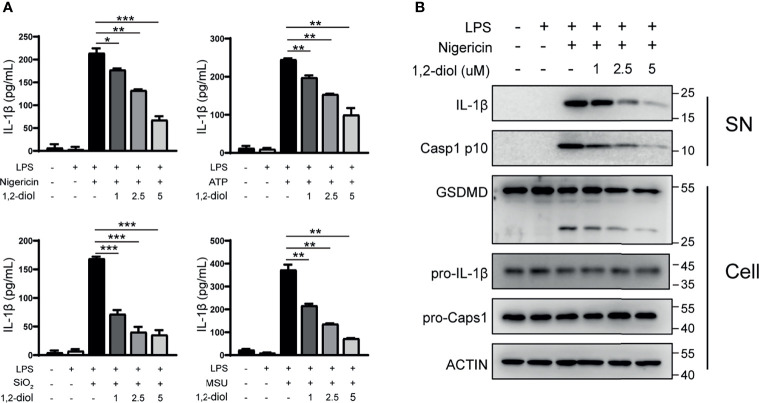
1,2-Diol suppresses NLRP3 inflammasome activation in THP-1 cells. **(A)** ELISA of IL-1β in supernatant from PMA-primed THP-1 cells treated with LPS stimulation, after that the cells were treated with or without different doses of 1,2-diol and stimulated with ATP, nigericin SiO_2_ or MSU. **(B)** Western blots for Caspase-1 and IL-1β in the supernatant or the cleavage of GSDMD, the expression of pro-Caspase-1 and pro-IL-1β in the cell lysates of PMA-primed THP-1 cells treated with LPS stimulation, after that the cells were treated with or without different doses of 1,2-diol and stimulated with nigericin. (*p < 0.05; **p < 0.01; ***p < 0.001).

Next, we examine whether 1,2-diol can suppress NLPR3 inflammasome in human cells. Consistent with mouse peritoneal macrophages, after treating 1,2-diol with PMA-primed THP-1 cells and challenged with the above-mentioned agonists, IL-1β secretion was dose-dependent inhibited (**Figure 2A**). Western blot analysis revealed that 1,2-diol suppressed the activation of Caspase-1 and IL-1β in the supernatant, and attenuated the cleavage of GSDMD but did not impair the precursors of IL-1β and Caspase-1 (**Figure 2B**). Together, these results indicated that 1,2-diol also inhibits NLRP3 inflammasome in human cells.

### 1,2-Diol Inhibits ASC Oligomerization and Inflammasome Assembly

**Figure 3:**
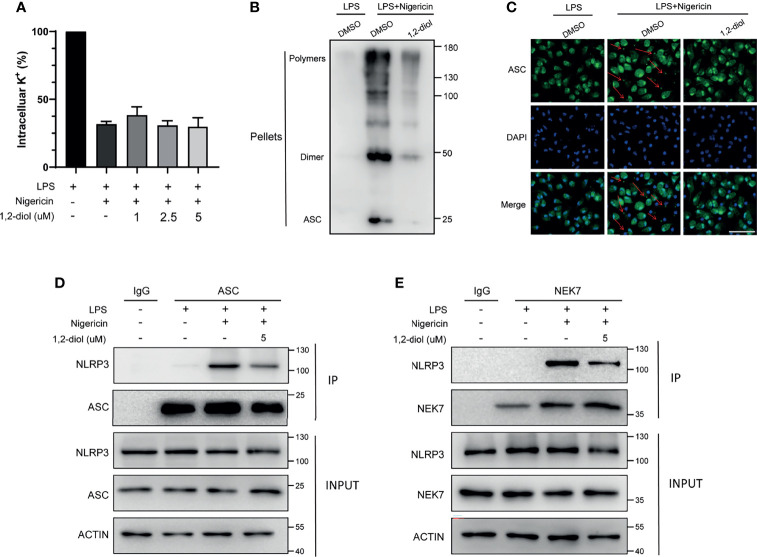
1,2-diol inhibits ASC oligomerization and inflammasome assembly **(A)** Qualification of potassium efflux in LPS-primed peritoneal macrophages treated with different doses of 1,2-diol and then stimulated with nigericin. **(B)** Immunoblot analysis of ASC oligomerization in the cell lysates of LPS-primed peritoneal macrophages treated with 1,2-diol and then stimulated with nigericin. **(C)** Immunofluorescent staining of ASC and representative images of ASC speck. **(D)** IP and immunoblot analysis of the interaction of NLRP3 and ASC in the cell lysates of LPS-primed peritoneal macrophages treated with 1,2-diol and then stimulated with nigericin. **(E)** IP and immunoblot analysis of the interaction of NLRP3 and NEK7 in the cell lysates of LPS-primed peritoneal macrophages treated with 1,2-diol and then stimulated with nigericin.

We next investigated how 1,2-diol inhibited NLRP3 inflammasome activation. It has been proposed that potassium (K^+^) efflux is a trigger common to several NLRP3 activators, namely, nigericin, ATP, MSU and SiO_2_. So we tested whether 1,2-diol could block potassium. The results confirmed that 1,2-diol could not block nigericin-induced potassium efflux (**Figure 3A**), indicating that 1,2-diol does not affect the potassium efflux during NLRP3 inflammasome activation.

Since ASC oligomerization and the formation of ASC speck are essential steps for NLRP3 activation, we next examined the formation of nigericin-induced ASC oligomers and ASC speck. The results demonstrated that 1,2-diol could significantly suppress the formation of ASC oligomers (**Figure 3B**) and could reduce the percentage of cells containing ASC speck (**Figure 3C**), suggesting that 1,2-diol may affect the ASC oligomerization or the upstream of ASC oligomerization to suppress the NLRP3 inflammasome activation.

Next, we assessed whether 1,2-diol could impact the NLRP3-ASC interaction which is a critical step for ASC oligomerization and NLRP3 inflammasome. The results showed that 1,2-diol inhibited the endogenous interaction between NLRP3 and ASC in nigericin-treated macrophages (**Figure 3D**). Thus, these results investigate that 1,2-diol could block NLRP3-ASC complex formation to inhibit ASC speck formation and NLRP3 inflammasome activation.

Recently, NEK7 has been proposed as an essential component of NLRP3 inflammasome and NEK7–NLRP3 interaction is important for subsequent NLRP3 inflammasome assembly and recruitment of ASC to NLRP3 (11). So we used immunoprecipitation and immunoblotting assays to verify whether 1,2-diol could suppress the endogenous interaction between NEK7 and NLRP3 promoted by nigericin. The results showed 1,2-diol could markedly prevent the interaction of NEK7 and NLRP3 (**Figure 3E**). Thus, these results suggest 1,2-diol may block ASC speck formation and NLRP3 inflammasome assembly by preventing NLRP3–NEK7 interaction.

### 1,2-Diol Was Readily Docked With NLRP3

**Figure 4:**
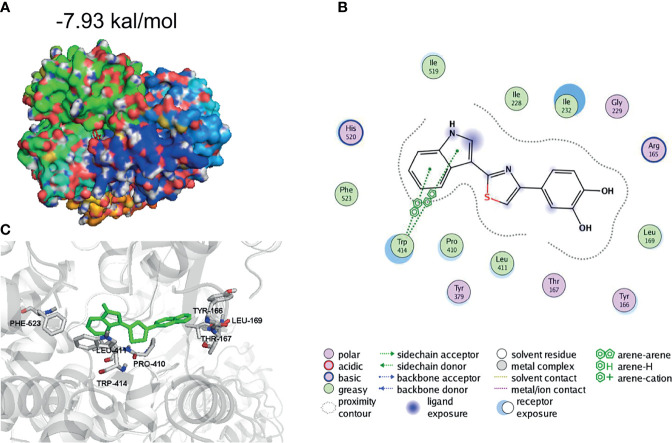
1,2-Diol was readily docked with NLRP3. **(A, B)** 3D binding mode diagrams between NLRP3 and the chemical 1,2-diol. The protein is shown in cartoon and colored in gray, key amino acid residues were shown as sticks. Docking score is −7.93kcal/mol. **(C)** 2D mode diagrams the key amino residues in the ligand−protein complexes.

To further evaluate how 1,2-diol inhibited NLRP3 inflammasome, we performed molecular interactions of 1,2-diol and template structure of NLRP3 (Protein Data Bank accession no. 6NPY) by AutoDock4. After calculating, the results showed that 1,2-diol was readily docked into the NLRP3 pocket with −7.93 kcal/mol binding energy (**Figure 4A**). The interaction residues of NLRP3 with 1,2-diol including PHE523, LEU411, PRO410, TRP414, TYR166, LEU169, and THR167 (**Figures 4B, C**). Thus, these results suggest that 1,2-diol may have an affinity binding site to the NLRP3 inflammasome.

### 1,2-Diol Inhibits NLRP3 Activation *In Vivo* and has Beneficial Effects in LPS Induced Mouse Models of ALI

**Figure 5:**
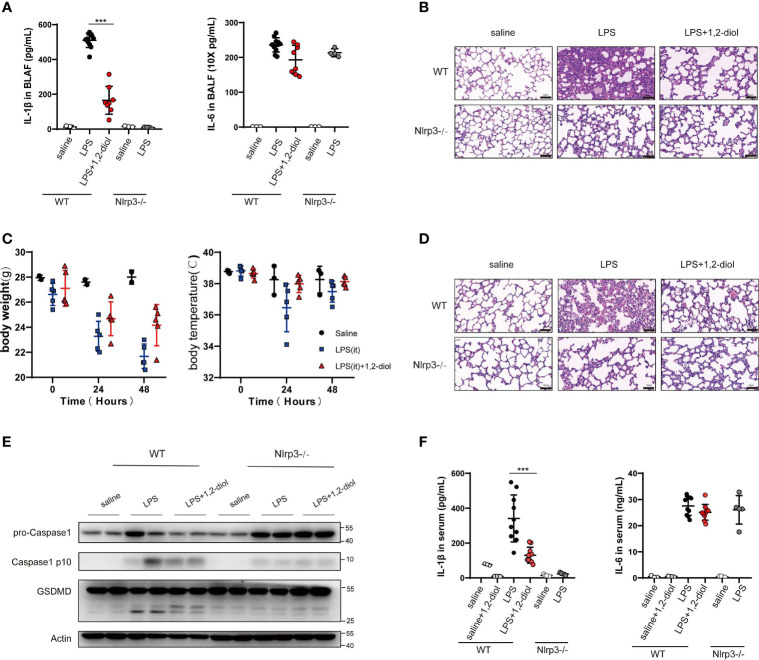
1,2-Diol inhibits NLRP3 activation *in vivo* and has beneficial effects in mouse models of LPS induce ALI. **(A)** IL-1β and IL-6 release in the BLAF were measured by ELISA. WT or Nlrp3^−^/^−^ mice were pretreated with 40 mg/kg, 12-diol by intraperitoneal injection 30 min before i.t. instilled with 15 mg/kg LPS. **(B)** H&E staining show representative images of lung in WT or Nlrp3^−^/^−^ mice which i.t. instilled with 15 mg/kg LPS (scale bar, 50 μm). **(C)** The body weight and the body temperature were recorded during ALI. 1,2-diol treatment has a tendency to reduce the loss of body temperature and body weight. **(D)** H&E staining showed representative images of lung in WT or Nlrp3^−^/^−^ mice which was intraperitoneal challenged with LPS 25 mg/kg (scale bar, 50 μm). **(E)** Western blots for the cleavage of GSDMD and the expression of Caspase-1 in lung. WT or Nlrp3^−^/^−^ mice were pretreated with 40 mg/kg 1,2-diol by intraperitoneal injection 30min before intraperitoneally challenged with LPS 25 mg/kg. **(F)** IL-1β and IL-6 released in the serum were measured by ELISA. WT or Nlrp3^−^/^−^ mice were pretreated with 40 mg/kg 1,2-diol by intraperitoneal injection 30 min before intraperitoneally challenged with LPS 25 mg/kg. (***p <0.001).

We next examined whether 1,2-diol could inhibit NLRP3 activation *in vivo* and has beneficial effects in mouse models of LPS induce ALI. We first induced ALI by intra-tracheal delivery LPS.The results showed that the level of IL-1β in the lung BALF was significantly decreased after 1,2-diol treatment and IL-6 was only slightly decreased (**Figure 5A**). Furthermore, we observed a marked decreased ALI pathology by treating 1,2-diol (**Figure 5B**). Meanwhile, the body weight and temperature of mice were detected every 24 h with comprehensive, detailed records and statistics (**Figure 5C**). To further investigate whether 1,2-diol could inhibit NLRP3 inflammasome *in vivo*, then we induced endotoxemia model by i.p. injection of LPS. We observed a marked decrease in sepsis-related lung injury by 1,2-diol treatment as evaluated by the histopathology (**Figure 5D**). In addition, we detect the cleavage of GSDMD, the pro-Caspase-1 and cleavage Caspase-1 in lung tissues. Treatment of 1,2-diol significantly inhibited the cleavage of GSDMD and Caspase-1 in lung tissue in endotoxemia mice (**Figure 5E**). At last, we found that the level of IL-1β in the serum was significantly decreased by treatment with 1,2-diol in endotoxemia mice (**Figure 5F**). Overall, 1,2-diol inhibits NLRP3 activation *in vivo* and has beneficial effects in mouse models of LPS induce ALI.

## Discussion

As a severe respiratory inflammatory syndrome, ALI is considered to be one of the most important causes of morbidity and mortality in critically ill patients (19, 20). Destruction of the alveolar epithelium, exudation of protein-rich liquid into the alveoli and related external factors such as the collapse of lung tissues jointly caused and exacerbated the development of ALI (21). Meanwhile, infectious etiologies, such as sepsis and pneumonia, are also the significant catalysts and leading causes of ALI/ARDS (22). ALI not only has serious consequences for general health, but also is one major contributor to intensive care unit (ICU) costs. Despite the treatment strategies have some marked improvements in the delivery of critical care, the mortality of ALI still remains high, with no new and effective pharmacologic therapies or alternative medicine approved (23).

In the present study, we used i.t. administration of LPS, the component of the Gram-negative bacterial cell membrane which has been widely used to induce pulmonary inflammation in animal models of severe lung injury to induce ALI, with characteristic symptoms, namely, pulmonary edema, intrapulmonary hemorrhage and excessive leukocyte accumulation (15). Here we have surprisingly found a novel small molecule compound 4-benzene-indol derivative (1,2-diol), ameliorated LPS-induced sepsis-related ALI by inhibiting the NLRP3 inflammasome.

NLRP3 inflammasome is a multiple-protein complex that in humans is encoded by the NLRP3 gene, which is located on the long arm of human chromosome (24). NLRP3 inflammasome is expressed preponderantly in macrophages and could trigger an array of immune responses after being activated (25). Because the immune system is the very first body system to be affected by sepsis and the indispensable role played by NLRP3 inflammasome, a series of approaches have been researched to modulate it in the initiation, progression, and clinical therapy. Some pieces of evidence had already demonstrated that NLRP3 inflammasome activation could induce significant changes in different body systems affected by sepsis, among which, the effects of NLRP3 inflammasome on the respiratory system includes immunological influences like edema formation, neutrophil infiltration, and elevated levels of inflammatory cytokines such as IL-1β and IL-18 in the lung tissue (26). Meanwhile, the therapeutic efficacy on respiratory system of targeting the NLRP3 inflammasome pathway has been extensively studied and has some practical applications (27). For example, the administration of cinnamaldehyde, a phenolic compound from Cinnamon species, can reduce the expression level of IL-1β and NLRP3 in the lungs of LPS-injected mice (28). Another study demonstrated that hemin (an inducer of HO-1) was able to decrease the expression of ASC, Caspase-1 and NLRP3, the activation of NLRP3 inflammasome could also be weakened (29). The protective effects for CLP-induced acute lung injury of mice by using dihydromyricetin have also been proved which could inhibit the activation of NLRP3 inflammasome then reduce pyroptosis (30). Up to the present, molecular mechanisms and related signaling pathways indicate that preventing the assembly and activation of NLRP3 inflammasome could reduce the inflammatory response in sepsis.

**Figure 6:**
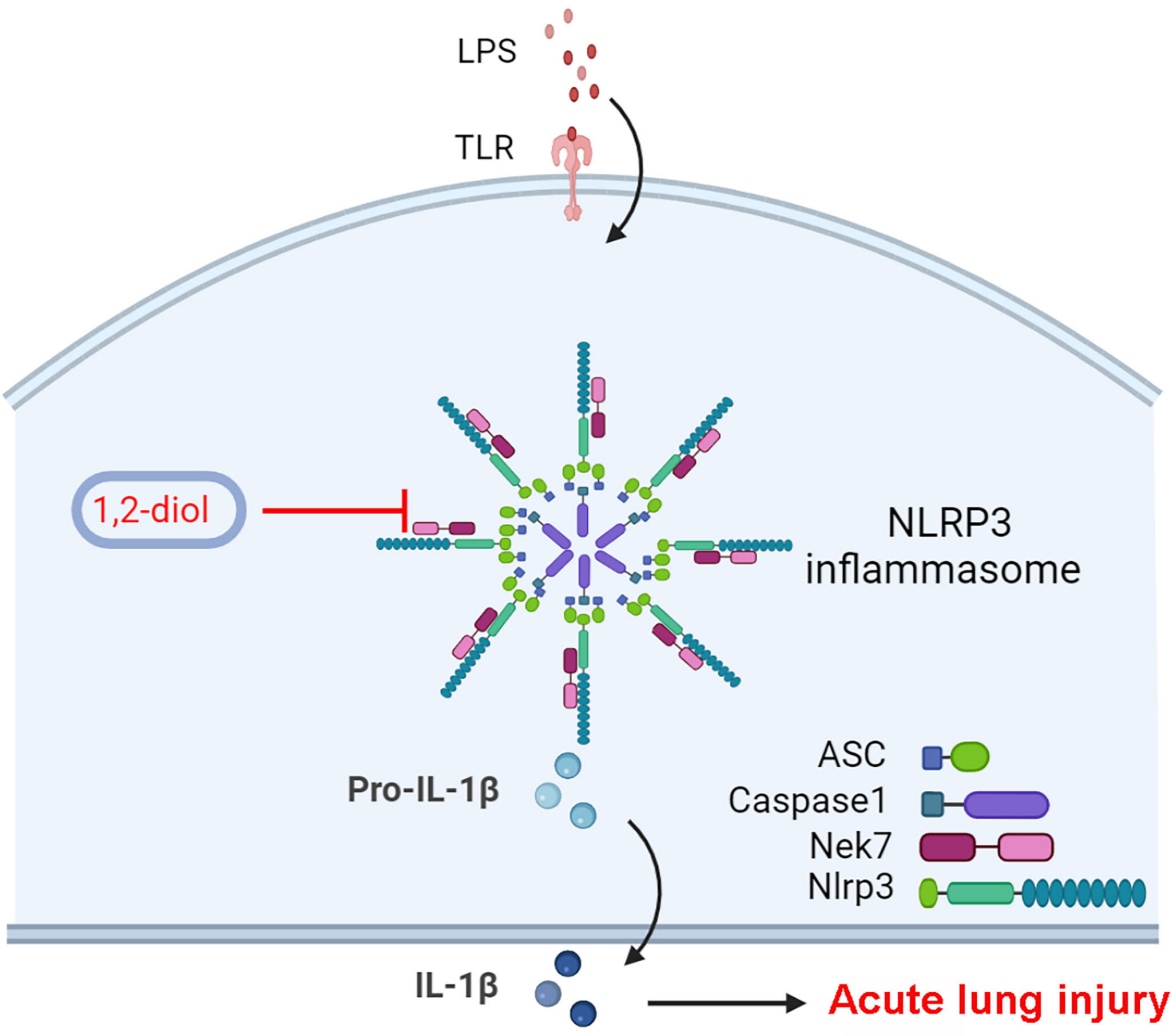
Schematic diagram of suppressive role of 1,2-diol on NLRP3 inflammasome.

In this research, a conspicuous discovery of treatment-related translational use for a 4-benzene-indol derivative (1,2-diol) was elaborated by screening a large number of chemical compounds. The results showed that 1,2-diol could not only suppress the activation of NLRP3 inflammasome both *in vitro* and alleviate LPS-induced ALI *in vivo*, but also suppress the NLRP3 inflammasome in human myeloid leukemia mononuclear cells (THP-1) cell lines. Mechanistically, we found out that the inhibition effect of 1,2-diol did not depend on the potassium (K+) efflux, which has been well accepted as an essential process both necessary and sufficient for NLRP3 inflammasome activation, because of the stable results of intracellular potassium concentration. However, the combination of NLRP3 and ASC was significantly affected. It has been widely confirmed that NLRP3 inflammasome is composed of the sensor protein NLRP3 connected to Caspase-1 through the adaptor protein ASC, upon inflammasome activation functional oligomeric inflammasome particles of NLRP3 and ASC were released from cells, acting as danger signals to amplify inflammation by promoting the activation of Caspase-1 extracellularly (25, 31, 32). Our immunoprecipitation (IP) experiment also showed that 1,2-diol could affect the combining of NLRP3 and ASC. Further exploration of upstream mechanism has shown that the combination of NLRP3 and NEK7 was markedly inhibited, since NEK7 is considered as an essential mediator of NLRP3 activation downstream of potassium efflux. Therefore, the abovementioned experimental results comprehensively demonstrate that 1,2-diol firstly affected the combination of NEK7 and NLRP3, then affected the expression of the downstream target ASC and thus affecting the activation of NLRP3, thereby influencing the splicing of Caspase-11 as well as the release of IL-1β. The possible molecular targets were further predicted by software which showed that NLRP3 might tightly linked to 1,2-diol **(****Figure 6****, created with BioRender)**. Collectively, 1,2-diol blocks the NLRP3 inflammasome activation by disrupting NLRP3-NEK7 interaction and the subsequent NLRP3 inflammasome assembly and activation. 1,2-diol may serve as a potential therapeutic drug and NLRP3 inflammasome signaling would be a novel pharmaceutical target for clinical treatment of sepsis-related ALI. The pieces of evidence presented here demonstrated that inhibiting the assembly and activation of this NLRP3 inflammasome could prevent the inflammatory response normally visualized in sepsis-related ALI. Still, further studies on 1,2-diol are necessary to demonstrate the detailed influence exerted by the NLPR3 inflammasome on sepsis pathophysiology and to design new treatment approaches such as decreasing toxicity and increasing efficacy, pharmaceutical and related translational research.

## Conclusions

Overall, our research provides a new promising therapeutic drug 4-benzene-indol derivative (1,2-diol) in avoiding the establishment of sepsis-related ALI. The 1,2-diol marks a new pharmacological approach for treating NLRP3 inflammasome-related diseases, whereas the effectiveness in mankind cases clinically requires more investigations nonetheless.

## Data Availability Statement

The original contributions presented in the study are included in the article, further inquiries can be directed to the corresponding authors.

## Ethics Statement

The animal study was reviewed and approved by the Central South University.

## Author Contributions

YB, JL and JS contributed to study design. JL, YB and JS conducted animal experiments. YZ, XW, LL and ZL performed data analysis. YB, JS and MC wrote the original manuscript. YZ and YT edited the manuscript. JS, JL and YB made the revision. All authors listed have made a substantial, direct, and intellectual contribution to the work and approved it for publication.

## Funding

This research was supported by the National Natural Science Foundation of China (Nos. 81971893, 81700127), the Excellent Postdoctoral Program for Innovative Talent of Hunan (2021M693564), the China Postdoctoral Science Foundation (2020TQ0364) and Fundamental Research Funds for the Central Universities of Central South University (2021zzts0405).

## Conflict of Interest

The authors declare that the research was conducted in the absence of any commercial or financial relationships that could be construed as a potential conflict of interest.

## Publisher’s Note

All claims expressed in this article are solely those of the authors and do not necessarily represent those of their affiliated organizations, or those of the publisher, the editors and the reviewers. Any product that may be evaluated in this article, or claim that may be made by its manufacturer, is not guaranteed or endorsed by the publisher.
